# Challenges and opportunities for improving mental health care and preventing suicide among people living with HIV: Perspectives of mental health professionals in Tanzania

**DOI:** 10.1371/journal.pgph.0002762

**Published:** 2024-02-16

**Authors:** Elizabeth T. Knippler, Alyssa J. Martinez, Ismail Amiri, Kim Madundo, Blandina T. Mmbaga, David B. Goldston, Michael V. Relf, Brandon A. Knettel

**Affiliations:** 1 Duke Center for AIDS Research, Durham, North Carolina, United States of America; 2 Duke University School of Nursing, Durham, North Carolina, United States of America; 3 Duke Global Health Institute, Durham, North Carolina, United States of America; 4 Kilimanjaro Christian Medical Centre, Moshi, Tanzania; 5 Kilimanjaro Christian Medical University, Moshi, Tanzania; 6 Kilimanjaro Clinical Research Institute, Moshi, Tanzania; 7 Department of Psychiatry & Behavioral Sciences, Duke University, Durham, North Carolina, United States of America; University of Auckland, NEW ZEALAND

## Abstract

People living with HIV (PLWH) experience unique stressors that contribute to emotional distress, and PLWH are more than twice as likely to die by suicide when compared to the general population. In countries like Tanzania, there is a relatively high burden of HIV but few resources to support mental health needs. To gain a better understanding of mental health challenges experienced by PLWH in northern Tanzania and identify opportunities for intervention, we interviewed 12 mental health professionals working in the Kilimanjaro region. Thematic analysis was used to explore drivers and impacts of emotional distress, community influences on mental health, and gaps and barriers to existing mental health care. Perspectives from mental health workers highlight the compounding effects of stress related to HIV status, family conflict, finances, and other social challenges, which can lead to poor HIV treatment outcomes and suicidal ideation. Cultural beliefs and stigma surrounding both mental health and HIV limit care-seeking behavior for mental health issues. Those who do seek care often encounter barriers related to poor mental health infrastructure, including a lack of providers, limited financial resources, and little integration into other health services. There is a clear need for investment in the mental health care system, as well as interventions to improve knowledge and perceptions of mental health and comprehensively address stressors. We describe feedback on a proposed telehealth counseling intervention integrated into routine HIV services, which shows strong potential to mitigate barriers to mental health treatment, reduce suicidal ideation, and support the wellbeing of PLWH.

## Introduction

In 2021, an estimated 38.4 million people were living with HIV worldwide, with 1.5 million having become newly infected that year; 60% of these new infections occurred in Africa [1]. Despite the increased access to lifesaving antiretroviral treatments and subsequent improvements in long-term survival, many people living with HIV (PLWH) continue to face a considerable emotional burden related to their diagnosis [2–4]. PLWH are at increased risk for developing psychiatric disorders and may experience mental health challenges throughout their illness journey [5, 6]. The COVID-19 pandemic led to a disruption of HIV, AIDS, and mental health services in many settings, including Tanzania, which has exacerbated emotional challenges among the most vulnerable populations of PLWH [7, 8].

Several factors may contribute to mental health challenges among PLWH. Internalized shame and worry about the diagnosis, coupled with a lack of comprehensive disease knowledge and stereotypes about HIV, can lead to uncertainty about one’s future health, concerns about HIV transmission, and challenges in accessing and maintaining care [9–11]. Anticipation of potential stigma and discrimination from others, as well as the actual experiences of enacted stigma and mistreatment, may contribute further to stress, social isolation, and fears around disclosure of one’s HIV status [10, 12, 13].

Depression and suicidal ideation are highly prevalent among PLWH and are associated with significant increases in risk for suicide [2, 5, 14]. PLWH are more than seven times as likely to attempt suicide and more than twice as likely to die by suicide when compared to the general population [15–17]. When untreated, mental health challenges affect functioning and motivation across multiple areas of living, including family and social relationships, school or income-generating activities, and health [18, 19]. Mental health symptoms commonly have a negative impact on HIV care engagement, medication adherence, and transmission prevention behaviors, yet routine screening or diagnosis of common mental health challenges is rare in HIV care; therefore, it is critical to integrate mental health screening and treatment into HIV services [20].

Sub-Saharan Africa continues to be the region of the world most affected by the HIV pandemic. Rates of depression and suicidal ideation are exceedingly high among PLWH in Tanzania [2, 4, 6]. In a study conducted in the major Tanzanian city of Dar es Salaam examining 100 deaths by suicide, 26% were among PLWH [21]. In that study, death by suicide among PLWH was frequently associated with physical health challenges arising from avoidance or disengagement from HIV care [21]. In previous studies from the Kilimanjaro region, where the current study was conducted, 25%-41% of PLWH met criteria for depression [2, 4], and 13% of pregnant women living with HIV reported suicidal ideation [6]. However, in many sub-Saharan African countries such as Tanzania, there are few mental health professionals trained to meet the demand of co-occurring HIV and mental health challenges [22–24].

There is a clear and urgent need for improved mental health services in HIV care in Tanzania [20, 25]. In order to effectively prevent deaths and treat mental health conditions among PLWH, it is vital to understand the surrounding clinical, cultural, social, and political environments, including stigma related to both HIV and mental health. The objectives of this study were to improve our understanding of the unique mental health challenges experienced by PLWH in the Kilimanjaro region of Tanzania and gain insight into the services needed to address these challenges. To do this, we interviewed local mental health professionals, with the ultimate goal of informing intervention efforts.

## Methods

### Setting and sample

We conducted in-depth qualitative interviews with 12 mental health workers to inform the development of a brief telehealth counseling intervention to reduce suicidality and improve HIV care engagement in an urban setting in northern Tanzania. The chosen sample was appropriate for our study given the limited number of mental health professionals in this setting and our aims of achieving data saturation and generating rich, context-specific findings [26]. We recruited mental health workers who were familiar with mental health challenges among PLWH in Moshi, Tanzania, where adult HIV prevalence is approximately 4.8%.

The 12 participants were 43 years old on average (range 30–59 years) and included nine women and three men. Participants included four nurses working in mental health settings, three medical doctors (two psychiatrists and a pediatrician), two medical officers (equivalent to a physician assistant), one Master’s-level counselor, one social worker, and one Bachelor’s-trained mental health worker. Participants had been working in the mental health field between 1 and 25 years, with a mean of 9 years. Eleven identified as Black/African, and one identified as White.

In northern Tanzania, HIV care is provided free of charge with support from Tanzania’s National AIDS Control Program [27]; however, mental health screening and treatment is generally not incorporated into HIV care [25, 28]. Despite the successful decentralization of HIV care to many smaller clinics in Tanzania, mental health care in the country remains largely centralized at major hospitals, where suicide prevention has rarely been prioritized [22].

### Ethics statement

All participants provided written informed consent to participate in the study and gave permission to audio record the interview. Participants were informed that they could stop participation or decline to respond to a question at any time without penalty. Study staff communicated the sensitive nature of the topic and potential for emotional responses, asked participants about any concerns they had, and monitored for any signs of emotional distress during the interview.

A research team member conducted the interview in either Kiswahili or English, using a semi-structured interview guide. Interviews were conducted from November 2021 through January 2022 and lasted approximately 30 to 40 minutes. Participants were compensated with a small gift of appreciation valued at 10,000 Tanzanian shillings ($4.25 USD). Study procedures were approved by the ethical review boards of Duke University Medical Centre IRB (Pro00107424), the Kilimanjaro Christian Medical Centre IRB (Protocol 1307), and the Tanzanian National Institute for Medical Research (NIMR) (Protocol NIMR/HQ/R.8c/Vol.I/2122).

### Procedures

Potential participants were first identified through the psychiatry units at two tertiary hospitals, including one regional referral hospital and one zonal referral hospital. Mental health professionals were approached at their place of work and asked if they were interested in completing an interview about how to best support the mental health needs of PLWH. Upon completion of the interview, participants were asked to identify other mental health workers in local settings who might be eligible to participate, and these professionals were then contacted.

The semi-structured interview guide included prompts to explore the participant’s professional role, care offered at the clinic, and mental health challenges common among PLWH. The interviews included questions about HIV and mental health stigma, availability of emotional support, and the impacts of mental health challenges on HIV care. The interviewers asked about suicidality among PLWH and explored opportunities for future interventions, including by eliciting feedback on a proposed model for a telehealth counseling intervention.

In the proposed model for a counseling intervention, HIV nurses would routinely screen all patients at their clinic for suicidal ideation. Identified patients would then be connected to nurse counselors based at a different facility via a video call. Interview participants were asked to express their opinions of the intervention model, their thoughts on ideal counseling session content and format, and potential foreseen challenges and benefits of using telehealth. The interviewers probed for potential acceptability and feasibility of a telehealth model among HIV clinic staff, counselors, and patients in the setting and solicited recommendations to help inform refinement of the intervention [29, 30].

### Data analysis

Audio recordings were de-identified and transcribed, and those conducted in Kiswahili were translated into English. The transcripts were analyzed in NVivo 12 software [31] using an applied thematic approach [32, 33]. To ensure credibility and trustworthiness of the findings, the coding team represented both Tanzanian- and U.S.-born study team members. We recognize the legacies of colonialism that have shaped the fields of global health, mental health, and HIV care, and strive to be aware of our identities, privileges, and biases and the ways they shape our approach to research. We are intentional in our efforts to attend to the power dynamics inherent in global north-south partnerships and to foster equity within our research teams. We believe in the value of having multiple individuals as part of our coding team who come from different backgrounds. Team members engaged in a collaborative and iterative process of analysis that allowed us to reflect on our positionality and minimize bias in the analysis of the data.

An initial codebook was developed collectively using the interview guide and emergent themes from an immersive read-through of the transcripts. Three team members then independently coded two transcripts and reviewed the coding together to further refine the codebook and ensure consistency in how codes were interpreted and used. The remaining transcripts were coded by two independent coders, and any discrepancies were discussed and resolved to consensus. By the end of analysis, few new themes emerged, confirming that the data had approached saturation. Queries were run to pull the coded sections from the transcripts for each of the main domain codes and sub-codes, and to identify representative quotations to describe the patterns of responses among participants.

## Results

Interviews with the 12 mental health workers provided insight into the landscape of stressors, mental health challenges, available care and barriers to care, community influences on mental health, and potential opportunities to support the mental health needs of PLWH in northern Tanzania.

### Perceived contributors to emotional distress for PLWH

**Fig 1:**
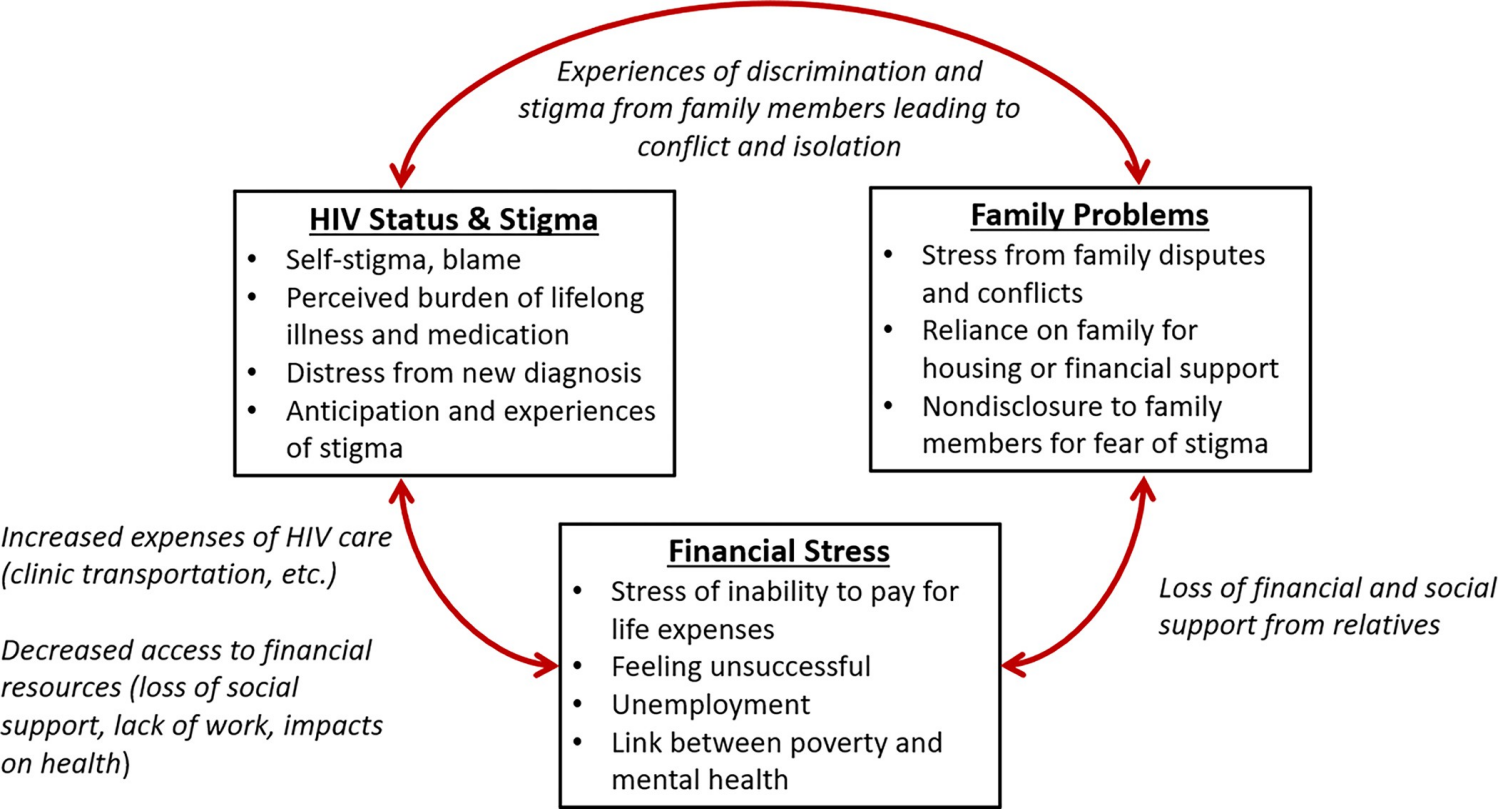
Contextual factors related to emotional distress and hopelessness among PLWH.

The mental health professionals described three main categories of stressors that they believe most frequently contribute to mental health challenges among PLWH: their HIV status and associated stigma, family problems and other relational challenges, and financial stress. Participants commonly described interconnections among these stressors, which can lead to a compounding of challenges for PLWH (see Fig 1).

Mental health workers noted the unique stressors experienced by PLWH due to their HIV status, including experiences of stigma and discrimination from others, feelings of internalized stigma, and the overwhelming sense of living with a chronic medical condition. Participants shared that HIV is highly stigmatized in their community and still erroneously viewed by some as an untreatable disease resulting in premature death. One participant pointed to stigma as the likely explanation for high rates of suicide among PLWH:

Having to face your family and tell them you live with HIV, or face your partner and tell them, or [experience] fear of getting fired from your job… [or] just all the societal ramifications due to stigma that are in the society. I’m certainly not surprised [that suicide is common]. And you know, with stigma, it almost always interplays with mental health.–Medical doctor

Several participants described HIV as a perceived hindrance to one’s future goals and dreams, which can lead to feelings of hopelessness. A psychiatrist described patients expressing “my dreams are over… my life span is shorter” and sharing their concerns about lifestyle changes and implications for their family and relationships:

[Given] the burden of taking medication and the side effects of medication, how will people look at me… what does that mean for my partner, for the future of my kids… what are the limitations in my social life and my work?

Participants described patients who felt self-blame or “self-stigma” related to their diagnosis, were slow to accept their HIV status, ruminated about how they had acquired HIV, or expressed feeling that they were “being punished” for poor decisions or past wrongdoing by being diagnosed with HIV.

Both HIV and mental health challenges carry stigma, which can affect a person’s willingness to disclose their challenges or prevent them from receiving social support. One nurse shared that in her experience, patients living with HIV “hardly recover” from psychiatric symptoms or conditions or “relapse more often,” attributing the difficult recovery to experiences of discrimination from relatives. Half of the participants named family problems, including isolation, exclusion, and stigma from relatives in response to HIV status, as a common cause of emotional distress for individuals living with HIV.

One participant noted the negative impact of internalized stigma on attitudes toward life, and the likelihood of subsequent mental health challenges, particularly among youth:

Depending on [the patient’s] condition, he is stigmatizing himself or giving up on himself. He feels as if he will not be able to achieve his goals… [Y]oung people have their future goals, so when he fails to achieve his dreams or goals, he becomes desperate and starts to think of suicide.–Social worker

This participant pointed to education about HIV, life skills, and entrepreneurship as key opportunities to reduce the mental health impact of an HIV diagnosis. One participant felt that “living conditions being very hard” was the main stressor for families that leads to suicidal thinking. Participants discussed social determinants of health such as monetary struggles, housing insecurity, and food insecurity as drivers of emotional distress by sharing stories of patients. A social worker described a young person who was struggling to afford food and school supplies and even contemplated dropping out of college before his needs were finally met: “The boy got frustrated and said ‘Why do I study when I don’t get food? I take my medicine when I am hungry.’” They described the importance of employment opportunities and the prospect of financial stability as catalysts for change in young people’s situations and attitudes.

Several participants described examples of familial conflicts unrelated to HIV, such as “fights among family members about land or property ownership.” In a setting in which resources are often shared among family members, rejection in response to one’s HIV status or mental illness can easily lead to increased financial hardship. A medical officer described one patient’s experience: “I think the real problem is financial. The family are not taking care of her, so she feels like maybe they are afraid of contracting HIV, that she can infect them… she’s just there alone.”

Participants discussed observations about other financial stressors, including the relationship between poverty and mental health challenges, inability to pay for expenses such as school fees or food, and mental health problems resulting from unemployment. HIV care is highly subsidized in Tanzania, but patients often incur costs related to transportation to their appointments and having to take time off from work. Participants noted that these financial burdens could exacerbate mental health challenges. As a medical officer explained, “Suppose those who want to commit suicide… are from [a neighboring district] and come here for antiretroviral drugs, and they don’t have money. That means they won’t be able to travel; at the end, it turns to mental illness.”

### Observed mental health challenges and impacts of stress

Mental health workers described seeing a variety of mental health challenges among PLWH resulting from the above stressors. Patients experiencing distress were often referred to patients by nurses at the HIV clinic to receive mental health support. A few participants observed that the patients who are referred are often women, but that men seem more likely to die by suicide. A medical officer attributed this disparity to “the tendency of women to be more open about the issues that they face compared to men, who tend to be more closed up. This also makes men more prone to suicide.” A psychiatrist shared their observation that men tend to “remain with their problem for a very long period before they talk to someone,” and that men more frequently use alcohol as a coping mechanism, which may further contribute to their mental health problems. All participants reported having seen or heard about patients with suicidal ideation; one nurse commented that suicide attempts “tend to be few” but that ideation was present among “many” patients.

When asked about the observed impact of mental health challenges on HIV care, a majority of participants discussed challenges that patients experience related to medication adherence. One psychiatrist summarized ways in which depression can contribute to noncompliance with antiretroviral therapy or other medications:

When I go back to that example of depression, normally a patient feels tired and loses energy. He might blame himself too much for the things which happened, the things which make him not feel the courage to go to the hospital again, to take his medication. And even if he has medicine, he might get negative feelings that it is better not to take [it], although he knows the importance of it. So, he may not attend his clinic appointments, and he may stop using medicine, and he also may stop taking care of himself.

Another medical doctor shared that in some instances, not taking HIV medication could be considered “another form when we think about suicide,” commenting that in staff discussions of mental health problems with adolescents living with HIV, “a lot of it [what staff observed] was about [patients] just deciding not to take medicine and knowing that eventually that will probably kill them.”

The connectedness of medication adherence, physical health, and mental health was highlighted by a counselor who noted that emotional challenges can contribute to poorer HIV outcomes among patients who are not virally suppressed: “Physically, stress affects the body’s immune system, so… the immune system will start to decline and increase the risk of getting other opportunistic infections.”

### Gaps in existing mental health care and barriers to care

The interviews shed light on several barriers to providing care and support for individuals with mental health challenges related to mental health infrastructure, cultural perceptions of mental health, and logistical barriers (described in Table 1).

**Table 1:** Summary of qualitative themes related to gaps and barriers to mental health care.

Theme	Sub-Theme	Representative Quotes
Mental health infrastructure	Limited mental health resources and workforce	“There are few doctors in Tanzania who specialize in psychiatry… because they usually say there is no money in psychiatry.” “Even for us as the staff, sometimes we used to be stigmatized by our core workers… So many nurses don’t want to work on this unit, because of the stigma.” “Although we might seem many compared to other facilities, we are not enough.”
	Poor linkage to specialized care	“If you are really depressed and going through all those hoops, I think it’s hard. There is not good integration between mental health and HIV services.” “There is very little referral from the clinical aspects that come to us, like straight reference from CTC to mental health. There are very few compared to what we expect from literature.”
Cultural perceptions of mental health	Stigma surrounding mental illness and HIV	“Another challenge is that if you say that you have a suicide clinic, you have already added stigma there. They will wonder, who wants to go to the suicide clinic?” “There is already a stigma with HIV… over the years, it’s become less and less stigmatized but then it’s still there. And then couple that with mental illness which is not spoken about and is also not screened routinely.”
	Lack of knowledge about mental health care	“Some people think, ‘If I use medications for a month and I am ok, I feel better… I don’t need to continue with medication,’ so they don’t come.” “Outside the hospital, there are much-needed programs to educate the general public about mental health challenges and what are the important signs to identify so that one can go for help.”
	Ideas about help-seeking	“Women are more open to their life challenges than men, and this helps them to go to see a doctor or psychologist. Men remain with their problem for a very long period before they talk to someone.” “One may feel this is a problem he can handle in his condition, though everything he does fails.”
Logistical barriers	Financial burden	“In the past, the government was helping pay for psychotropic medication, but now it has stopped, so we don’t see some of these patients attending the clinics because they can’t afford the bus fare, and in addition to that, they have to buy their medications.” “Suppose if they are coming from poverty, they are unable to get bus fare… If they have children, they should pay the school fees, and it’s hard for them to buy the drugs.”

Several participants mentioned that the small size of the mental health workforce constrains the number of services that can be provided and patients who can be treated, in addition to putting stress on the workers themselves. Lack of mental health awareness and inadequate screening within the healthcare system can hinder the recognition of mental health challenges among patients. When patients are identified, there are commonly delays in connecting them to mental health care. The referral process is burdensome to both providers and patients, preventing appropriate linkage to care and leaving patients without support.

Participants identified opportunities to improve linkage to psychiatry services from other areas of the healthcare system, including HIV care. One psychiatrist acknowledged that although connections between HIV care and psychiatry clinics exist “formally on paper,” in actuality, “the interaction is not good.” Navigating the referral system can be difficult and discouraging for patients experiencing mental health symptoms, as one medical doctor explained: “If you are really depressed and going through all those hoops, I think it’s hard. There is not good integration between mental health and HIV services.”

### Community influences on mental health

Participants described cultural beliefs and perceptions of mental illness that they witnessed in their community that impact patients’ experiences as well as their care-seeking behavior. One medical doctor described that “mental health issues are not commonly talked about” and that messages promoting mental health and emotional wellbeing are “still seen as a sort of foreign concept.” Furthermore, counselors and nurses shared that mental illness is often viewed as “not socially acceptable” or “embarrassing.” Perceptions of societal stigma may be reinforced by the fact that a suicide attempt is a criminal offense in Tanzania. As a result, individuals experiencing emotional distress often suffer in silence. As one counselor shared, individuals may be hesitant to talk about their problems to a healthcare worker, or even to friends or family, for fear that “people will laugh at [them]” or that they will be stigmatized.

Many participants discussed a common perception that mental health challenges are not medical in nature but rather something spiritual. As one medical doctor described:

Most mental illness is taken as a spiritual attack or a sort of spiritual or religious problem. It is given another meaning, as opposed to the biological meaning, so someone is more likely to go to the traditional healers or to faith leaders rather than go to a doctor. And [as] it’s given a spiritual meaning, there’s also that aspect of shame, that [belief that] “Okay, maybe I am under spiritual attack because I am not clean. I have done something wrong, and my ancestors are not happy with me.”

A nurse explained that a suicide attempt may be viewed by some people as a “curse of the family” and that families might respond to it differently depending on their knowledge about mental health: “Those issues concerned with culture are managed in the home. But for those who are aware of mental illness, [they] bring them to the hospital.”

Participants shared that stigma regarding mental illness is present even in the healthcare system and can be perpetuated by health care providers, which hinders the identification of symptoms and prevents patients from receiving adequate treatment. This may be especially true for mental health challenges among PLWH, as one psychiatrist explained:

There is already stigma associated with HIV. Over the years, it has become less stigmatized, but it’s still there. And then couple that with [the stigma of] mental illness, which is not spoken about and is also not screened routinely by healthcare providers.

A nurse disclosed that mental health professionals themselves can be stigmatized for their area of work, and that some nurses avoid working in the mental health field due to concerns about stigma from others: “Even for us, as the staff, sometimes we can be stigmatized by our co-workers.”.

### Feedback on a proposed telehealth counseling intervention model

Near the end of the interview, participants were asked to provide their feedback on the preliminary model of an intervention focused on screening for suicidality during routine HIV care and linking patients by video call (telehealth) to trained counselors at a larger hospital. Participants were optimistic about the potential for telehealth counseling to provide expanded access to mental health care for patients in need but were cautious about its implementation.

Participants saw potential for the proposed intervention to overcome barriers to care, particularly in relation to transportation and linkage to mental health care. A social worker shared that they felt the proposed intervention would “reduce costs and time because the patient gets care right away… [and] is able to get more than one type of treatment [i.e., for HIV and mental health] at a time.” Other participants echoed the value of telehealth to minimize transportation challenges.

Several participants felt that telehealth could provide an effective introduction to therapy as well as an opportunity to identify and refer patients with higher mental health treatment needs for additional in-person care as needed. One psychiatrist shared their thoughts on the importance of routine screening for mental health issues:

My initial reaction… is excitement, in the sense that I feel like it will raise awareness and will address a number of challenges when it comes to identification and linkage to care and support for individuals with mental health concerns, specifically suicide, in this clinic.

This participant also emphasized the importance of providing HIV nurses with high-quality training to screen patients in a sensitive manner, noting that such training would be critical to successful implementation of the intervention.

Most participants expressed that the intervention, including both routine screening and telehealth counseling, would be acceptable to most health care workers if accompanied by proper training and explanation. One psychiatrist offered a caution about the possible perception of the screening for suicidal ideation as an additional burden on nurses:

The nurses would probably say that [it] would cause more work… now they have to talk to someone about a patient, or they now have to discuss these new symptoms they picked up in addition to all of the other workload that is there.

Participants felt that the intervention would be well-accepted among patients who are more familiar with technology and may like the idea of receiving counseling without having to travel. They noted, however, that some patients might find it difficult to navigate the technology and would require some support in this area.

When asked about potential concerns with the intervention, participants commonly identified uncertainty regarding the privacy of telehealth counseling. They emphasized the importance of transparency and confidentiality in interactions with patients, and of ensuring that no one else would listen in on the call from the counselor’s side. Participants expressed that it would be critical that both the telehealth counselor and patient be in private rooms during the intervention to ensure patient comfort. One psychiatrist underscored the importance of providing clear and full information regarding permission to audio record sessions for purposes of counselor training and supervision so that patients could give informed consent.

When asked about the potential for patients to access telehealth calls from home, participants expressed that some patients would struggle with this option depending on the level of privacy available to them and their openness with others about their HIV status. One counselor shared that young people might be hesitant to connect from their homes:

I think they [young people] do not want people to know about their status… They don’t know who is going to talk to me… and if there are people watching them as well, so I think they will be unwilling to call from home. They will prefer to come… and they will say, “Ok, now let’s talk one to one.”

## Discussion

People living with HIV may experience an increased burden of mental illness which can impact both their overall well-being and HIV care continuum outcomes. Our findings add to previous literature on the negative effects of stigma and shame, including the dual stigma of mental illness and HIV, that contribute to much higher rates of suicide among PLWH [34–36]. Perspectives from mental health workers highlight the ways in which an HIV diagnosis can compound and exacerbate existing stressors and remove sources of support for PLWH. Observed cultural beliefs that mental illness is a spiritual in nature, shame surrounding mental illness and suicide, and gender norms may prevent many PLWH from seeking help for mental health challenges. These findings point to the need for advocacy and a shift in public dialogue to normalize talking about mental health and seeking support [37, 38]. Creating a supportive environment for PLWH struggling with mental health challenges must occur both at the community level, where changing norms may be most influential, and at the national level, where suicide must be decriminalized [39–42].

It is increasingly recognized that improving health equity and outcomes for those most at-risk for suicide requires attention to the social determinants of health (SDOH) [35, 43]. Our findings show clear interrelationships among poverty, HIV, and mental health challenges: Major stressors included income and housing insecurity, food insecurity, lack of financial resources exacerbated by stigma, and family conflict. Future interventions should assess for and address the SDOH and use a strengths-based approach to identify and improve natural social supports and community resources [41, 44]. Improving financial well-being is likely to have a positive downstream impact on mental health and HIV outcomes [38, 45].

Our interviews highlight the need for investment in mental health infrastructure, including additional facilities and an increased workforce, across Tanzania to improve the existing system [23]. Improved screening within routine care (e.g., HIV clinics) and a more streamlined referral process can improve identification of individuals with mental health challenges and connect them to needed care [20, 28]. The cost of care, which is a limiting factor for many individuals with mental health challenges, can be addressed through subsidized treatment and increased access to health insurance [46].

Telehealth presents a relatively low-cost opportunity for facilitating integration of mental health in HIV care and increasing accessibility of services to patients in a region where there is limited mental health workforce and infrastructure [47]. The use of a telehealth intervention has potential for helping patients overcome financial and transportation barriers to mental health services. There was support from the mental health workers interviewed for exploration of telehealth as well as excitement about its potential to improve access to mental health care in this setting; however, their feedback highlights the need to attend to concerns about confidentiality, ease of use of technology, and human resource capacity to prevent additional burden on already overtaxed health systems. Identifying provider suggestions and potential challenges during the formative phase of intervention development was critical to developing a responsive, feasible, and sustainable intervention model and protocol for piloting the intervention [29]; for example, the use of WhatsApp, a platform that is already familiar and in wide use in this setting, can reduce the burden of needing to learn new technology, and the task shifting model of screening and referral piloted by our team has demonstrated early indications of a promising approach [28].

Our study sample of providers in a single urban area of northern Tanzania may not be generalizable to all contexts. This analysis included the perspectives of mental health providers only, but our future work will elicit the perspectives of HIV clinic providers, including on the acceptability of screening and referral for telehealth, as well as health care administrators to obtain perspectives on scalability and implementation. Analysis is currently underway to summarize the perspectives of PLWH who were interviewed during this phase of the research, with an emergent focus on social determinants of health. Integration of findings from each of these groups will provide a more comprehensive understanding of the mental health challenges for PLWH in this region and the potential for our telehealth intervention to provide needed support.

## Conclusions

There is a compounding effect of burdens arising from HIV diagnosis, mental health challenges, family conflict, social challenges such as poverty, and associated stigmas which contribute to poor HIV treatment outcomes and concerningly high presence of suicidal ideation among PLWH in northern Tanzania. Unfortunately, the current mental health system is poorly equipped to address these challenges in part due to lack of trained providers and poor integration of mental health services with HIV care. There is a clear need for multilevel interventions addressing public knowledge of mental health, systems improvement, improved screening, and targeted treatment of mental health challenges. A telehealth-delivered counseling intervention integrated into routine HIV care may provide a strong option to overcome common treatment barriers and address the unique mental health needs of PLWH.
